# HIV prevalence and demographic determinants of condomless receptive anal intercourse among trans feminine individuals in Beirut, Lebanon

**DOI:** 10.7448/IAS.19.3.20787

**Published:** 2016-07-17

**Authors:** Rachel L Kaplan, Justine McGowan, Glenn J Wagner

**Affiliations:** 1 Department of Obstetrics, Gynecology, and Reproductive Sciences, Bixby Center for Global Reproductive Health, University of California, San Francisco, CA, USA; 2 Center for Research and Education on Gender and Sexuality, San Francisco State University, San Francisco, CA, USA; 3 Global Health Sciences, University of California, San Francisco, CA, USA; 4 RAND Corporation, Santa Monica, CA, USA

**Keywords:** trans feminine, transgender, Lebanon, Middle East and North Africa (MENA), URAI, CRAI, HIV prevalence

## Abstract

**Introduction:**

Growing evidence suggests increased HIV incidence in the Middle East and North Africa among “key populations.” To date, epidemiological data have not accurately included and measured HIV prevalence and risk among trans feminine individuals in the region. Through the lens of the Gender Affirmation Framework, we assessed demographic correlates of risk behaviour and the prevalence of HIV among trans feminine individuals in Lebanon.

**Methods:**

Long-chain referral sampling was used to recruit 53 participants for completion of a behavioural survey and optional free rapid HIV tests. Data were collected using interviewer-administered questionnaires. A multivariable logistic regression model was used to identify demographic determinants of HIV risk behaviour.

**Results:**

Fifty-seven percent of participants reported condomless receptive anal intercourse (CRAI) with male partner(s) in the last three months, 40% of whom reported not knowing the HIV status of the partner(s). Of the participants tested for HIV as part of the study or via self-report, four (10%) were HIV positive; 13 declined HIV testing. Forty percent of the sample had no prior history of HIV testing. A history of trauma such as sexual abuse/assault was reported by almost half of the participants (49%). Sixty-eight percent reported experiencing physical violence and 32% police arrest, because of gender identity or presentation. A staggering 98% reported having experienced gender identity or gender presentation-related discrimination. Sixty-six percent of the sample reported current sex work; sex work was correlated with CRAI but was not significant in multivariate analysis. In regression analysis, “openness”/“outness” about transgender identity at work or school was significantly associated with CRAI. Surprisingly, a history of sexual abuse/assault was negatively correlated with CRAI, suggesting the need for further inquiry.

**Conclusions:**

The results of this study provide implications for how to address sexual health among trans feminine individuals in Lebanon and the greater Middle East and North Africa region.

## Introduction

While most regions throughout the globe have successfully achieved declines in the incidence of HIV, the Middle East and North Africa (MENA) region is one of the only regions still witnessing an increase in new infections [1]. Within the MENA region, the likelihood of the development of concentrated epidemics has been identified in Egypt, Yemen, Oman and Lebanon among “key populations” including men who have sex with men (MSM) and transgender populations [2]. Although transgender women's risk of HIV infection has been identified as 49 times higher than members of the general population throughout the globe [3], epidemiological data have yet to include and measure HIV prevalence and risk among trans feminine individuals in MENA [2].

Most HIV research to date that has taken place in MENA has failed to distinguish between MSM and transgender women [2]; therefore, little is known about HIV risk behaviour in this population despite staggeringly high rates of risk and infection globally. Among trans feminine individuals in other settings, a range of evidence has been gathered about HIV risk behaviour. Rates of unprotected sex are high, ranging, for example, from 34–37% in the United States [4,5] to 68% in Nepal [6]. Previous research has identified factors associated with unprotected sex among transgender women including drug and alcohol use, education level, and discrimination [4]. Intense stigma, and the anticipation of experiencing it, can limit access to resources and opportunities such as healthcare, employment, and HIV prevention programmes [7,8]. Family support has been shown to positively impact regular condom use among young transgender women [9]. Additionally, HIV prevalence rates have been found to be higher among transgender women who engage in sex work than those who do not [10,11]. Transgender women have reported high rates of having never been tested for HIV, ranging, for example, from 42% in Ontario, Canada [12], to 50% in Bangkok, Thailand [13].

In a region that is often characterized by clear distinctions between binary male and female categories, any ideas or individuals whose identity or presentation obscures or challenges these distinctions are viewed as a problem and sometimes even as a threat to the established order [14]. Indeed, a recent population survey conducted in Lebanon found that 98% of respondents adhered to the idea of the sex and gender binary, rather than a spectrum [15]. Further, the vast majority of respondents (82%) felt the need to be able to identify someone's gender when first meeting [15]; this finding parallels the structure of the country's first language in that the “you” form in Arabic in gendered, meaning that the speaker must choose the feminine or masculine form of “you” in order to address someone. The availability and accessibility of hormones and surgeries in Lebanon's heavily privatized healthcare system has yet to be documented formally; conflicting reports exist about the legality of obtaining gender affirmation surgery in the country [16,17]. It is, however, established that genital surgery is the only way for transgender individuals to be able to change their legal identification cards in the region [16]. Media reports indicate that many trans feminine individuals in Lebanon face discrimination and harassment in most aspects of their lives, from law enforcement to business owners, and from family members to passers-by on the street [17]. Further, they often face financial vulnerability due to challenges in securing and maintaining employment because of discrimination and perceived inconsistencies between government-issued identification and gender presentation [16]. Indeed, the first HIV study conducted among trans feminine individuals in Lebanon found that physical, emotional and financial threats to safety are common and that these experiences could impact vulnerability to mental health symptoms and risky sexual behaviours [18], as has been documented in other settings [8]. A lack of financial stability may result in individuals engaging in sex work and agreeing to have condomless sex if customers are willing to pay more for sex without condom [18]. Although only very recently applied to transgender populations, minority stress theory suggests that members of sexual (and/or gender) minority groups experience identity- and membership-related stress that have negative health consequences [19].

The Gender Affirmation Framework [20] offers a model for understanding needs and access in relation to HIV risk. Gender affirmation [20], “the process by which individuals are affirmed in their gender identity through social interactions” (p. 675), is a framework developed for conceptualizing risk behaviour among transgender women of colour that uses intersectionality theories and conceptual tools that have emerged from research on stigma, eating disorders and HIV. Developed in the United States among transgender women of colour who live at the intersections of transphobia, racism and sexism, the Gender Affirmation Framework provides a potential lens for interpreting the risk behaviour and experiences of trans feminine individuals in Lebanon who live at the intersections of transphobia and sexism–in addition to cultural collectivism, which prioritizes the group and/or family over the individual. This is in contrast with more individualistic societies found, for example, in mainstream United States [21]. Trans feminine individuals are not alone in their need for gender affirmation, but unlike non-trans counterparts, the need may take a more primary role in identity development due to gender minority status [20]. Social recognition of gender identity might include being perceived as feminine, being spoken to with the correct name and pronoun, and generally being treated with respect. In the present study, the Gender Affirmation Framework offers clues about how gender affirmation needs and access might impact risk behaviour in the Lebanese context.

The purpose of this study was to measure and interpret demographic determinants, HIV prevalence and risk behaviour associations among trans feminine individuals in Beirut, Lebanon, as the first to do so in MENA. Here we use the term “trans feminine individual” to refer to people whose gender identity, expression or behaviour is different from those typically associated with their binary sex of male assigned at birth. By using “trans feminine individual” rather than “trans woman” or “transgender woman,” we aim for inclusion and to avoid restriction of usage to any specific form of gender embodiment or identification. Further, in Lebanon, not all trans feminine individuals identify as women [18].

## Methods

### Participants and recruitment

Formative qualitative research [18] covering topic areas of relationships, sexuality, stigma, sexual behaviour, HIV testing and perceived norms about HIV testing that engaged the community was conducted prior to the implementation of this quantitative study. Long-chain referral sampling [22] was used to recruit participants between May and December of 2012. The sample included 53 trans feminine individuals who met the eligibility criteria of having been assigned male sex at birth and identifying as a woman or trans feminine, being 18 years or older, being fluent in Arabic or English, and residing in the greater Beirut area. Lebanese community organizations working with trans feminine individuals and study consultants identified individuals to be designated as “seeds.” Five “seeds” were given four recruitment coupons to recruit eligible trans feminine peers in their social networks and received $10 for each peer recruit, with a maximum potential for recruiting four participants. The coupons were uniquely coded to link participants to their survey responses and for monitoring who recruited whom, and reimbursement of participants for recruitment of peers. Participants were instructed to give a coupon to eligible trans feminine peers who were interested in participating, and to inform the potential participant to call the study coordinator for coupon verification, eligibility screening, verbal consent procedures and scheduling of an interview.

The interviewer-administered surveys took place at one of the collaborating community organizations or at a neutral location preferred by the participant. Upon completing the survey, participants received $30. Health referrals were provided for any participants who expressed interest in medical and mental health services. Each participant was also offered free finger prick rapid tests for HIV, hepatitis B and syphilis, as well as pre- and post-test counselling. For participants with positive test results, referrals were given for a free confirmatory test. Participants returned for confirmatory test results, counselling and referrals for medical treatment, if warranted. Individuals gave oral consent prior to participation. Institutional Review Boards at the RAND Corporation and Lebanese American University reviewed and approved this study.

### Measures

The study's survey was administered with computer-assisted software in Arabic or English, according to the participants’ preference. A team of researchers and community members developed the survey in English; the survey was then translated into Arabic and back-translated to ensure accuracy and consistency in meaning.

#### Demographics

Demographic characteristics included age, religious affiliation, education level, monthly income, country of birth, household members and sexual orientation. For the purposes of this study, participants’ religious affiliations were categorized as “Muslim,” “Christian” or “no religious affiliation.” Sexual orientation was categorized as “gay,” “heterosexual” or “other,” which included responses of “bisexual,” other” and “don't know/uncertain.” Highest education level was categorized as: “did not complete high school,” “completed high school” and “completed some/graduated university.”

#### Sexual behaviour

Sexual behaviour measurements included age of sexual debut, incidence of insertive anal sex with a man within the last three months, incidence of sex with a woman within the last three months, current engagement in sex work (for income of any kind) and relationship status. Insertive anal sex, sex work and relationship status were measured by binary variables. Participants were asked how many times they had had condomless receptive anal intercourse (CRAI) in the past three months; this measure was used as the primary risk behaviour of interest as although condomless insertive anal intercourse carries risk, CRAI is associated with much higher transmission risk. A binary variable was created with any participant reporting one or more instances of CRAI as having had risky sexual behaviour. This binary variable was used as the outcome variable for this analysis.

#### HIV testing and healthcare access

In order to measure HIV testing, participants were asked whether they knew where to go for a free HIV test and whether they had (ever) been tested for HIV. For HIV testing history, survey responses of “within the last 6 months” and “between 6 and 12 months ago” were combined under the subcategory variable “tested within 12 months.”

#### Gender affirmation

Although measures related to Gender Affirmation were not quantified as such because formal measures were not available at the time of data collection and the model had not been used in MENA, six variables were selected as relevant to the Gender Affirmation Model by two of the authors (RK, JM) who reviewed existing literature for applicability and pertinence [20]. Participants were asked about current hormone use for medical transition and whether they had had any gender confirmation surgeries. Self-reported satisfaction with one's body was measured on a five-point Likert scale; a binary variable was then created with the combined responses of “strongly agree” and “somewhat agree” compared to the combined responses of “uncertain,” “somewhat disagree” and “strongly disagree.” Comfort with gender was also measured on a five-point Likert scale and compiled into three subcategories: (1) “very comfortable” and “somewhat comfortable,” (2) “very uncomfortable” and “somewhat uncomfortable,” and (3) “uncertain.” Participants were also asked to rate their level of “openness” or “outness” as trans; one variable measured this in the context of participants’ personal/social lives and the other measured this in the context of the workplace or school. Responses were analysed in three categories: (1) “not at all,” (2) “a little bit,” “some” and “mostly,” and (3) “completely ‘out’ or ‘open’.”

#### Stigma and discrimination

A dichotomous discrimination variable was created to capture any “Yes” response to nine questions on experiences with discrimination that the participant perceived was due to trans identity or gender presentation. Family support for trans identity was measured by the survey question, “How supportive do you feel your family is regarding your transgender identity?” with response options provided on a six-point scale of 1 (“not at all supportive”) to 6 (“very much supportive”). History of arrest due to trans identity or gender presentation was measured by a binary variable, as was having experienced trans- or gender-related discrimination by a healthcare provider.

#### Trauma

Measures of trauma included having experienced physical and/or sexual abuse. A history of identity-related physical abuse was derived from a dichotomous question about whether the participant had been physically abused and thought it was because of their trans identity or gender presentation. A history of sexual abuse was measured via a “yes” response to having ever been forced or pressured into sexual activity.

#### Mental health

Mental health measures included depression, and suicidal ideation and attempts. Depression was measured by the 9-item Patient Health Questionnaire (PHQ-9) to assess the presence of depressive symptoms over the past two weeks. Participants were asked if they had “ever thought about or attempted suicide” to measure suicidal behaviour.

### Data analysis

Descriptive statistics derived from survey responses included measures of central tendency and variability for continuous variables and frequencies and percentages for categorical variables. For all tests of association, statistical significance was represented by *p* values less than 0.10. The χ^2^ test was used to analyse the association between reported CRAI within the last three months and selected categorical variables. Fisher's exact tests were used for categorical variables with less than five observations in any one of its categories. Wilcoxon rank-sum tests were conducted for variables with continuous measures. Using the results of these tests, a bivariate regression analysis was performed to further examine variables which were significantly associated with CRAI at the *p=*0.10 level, as well as other characteristics or behaviours with high prevalence in the study sample. The independent variables used in the bivariate analysis included measures of highest education level completed, transactional sex, monthly income, depression, suicidality, being “open”/“out” as a transgender individual at work or socially, family not at all supportive of transgender identity, history of arrest because of transgender identity and history of sexual abuse. To identify characteristics independently associated with CRAI, a multivariable model was constructed which included variables that were statistically significant in bivariate analysis.

## Results

### Demographics

Fifty-three trans feminine individuals were included in the analysis; one of the participants from the sample was excluded from the present analysis due to missing data for the outcome variable (CRAI). The median age of the sample was 22 years, with a range of 18 to 58 years. Most participants (89%) had been born in Lebanon and just over half (54%) of the sample identified as Muslim. While 40% of participants identified as heterosexual and 17% identified as gay, 43% had other responses including “bisexual,” “other,” and “don't know/uncertain.” Only three participants had graduated from university and over half (57%) had not completed high school. Forty percent of participants reported a monthly income of less than $500, while almost one third (31%) reported over $1000. The majority (68%) of participants reported living with someone, most of whom (58%) reported living with family member(s). None of the demographic characteristics were found to be significantly associated with having had CRAI in the last three months. Table 1 summarizes selected demographic characteristics of the sample (stratified by CRAI in the past three months).

**Table 1:** Demographic characteristics stratified by condomless receptive anal intercourse (CRAI) in the last three months

	Total population (*N*=53)	CRAI (*N*=30)	No CRAI (*N*=23)	
				
Characteristic	*n* (%)	*n* (%)	*n* (%)	*P*
Demographics				
Age, median (IQR)	22 (20, 30)	23.5 (20, 30)	22 (19, 35)	0.40
Religion				0.48
Muslim	28 (53.9)	15 (51.7)	13 (56.5)	
Christian	15 (28.9)	11 (37.9)	4 (17.4)	
No religious affiliation	9 (17.3)	3 (10.3)	6 (26.1)	
Highest education level				0.16
Did not complete high school	30 (56.6)	20 (66.7)	10 (43.5)	
Completed high school	14 (26.4)	5 (16.7)	9 (39.1)	
Completed some/graduated university	9 (17.0)	5 (16.7)	4 (17.4)	
Monthly income >$1000 USD	16 (30.8)	11 (36.7)	5 (22.7)	0.28
Born in Lebanon	46 (88.8)	26 (86.7)	20 (87.0)	0.98
Living with someone	36 (67.9)	19 (63.3)	17 (73.9)	0.41
Sexual orientation				0.16
Gay	9 (17.0)	5 (16.7)	4 (17.4)	
Heterosexual	21 (39.6)	15 (50.0)	6 (26.1)	
Other	23 (43.4)	10 (33.3)	13 (56.5)	
Sexual behaviour				
Age of sexual debut with male, median (IQR)	13 (10, 15)	13 (11, 16)	13 (9, 15)	0.25
Insertive anal sex with a male (in the past three months)	19 (35.9)	13 (43.3)	6 (26.1)	0.19
Sex for income	35 (66.0)	24 (80.0)	11 (47.8)	**0.01**
Relationship status: currently in a committed relationship	19 (35.9)	12 (40.0)	7 (30.4)	0.47
HIV testing and health care access				
Tested for HIV				0.54
Never	21 (39.6)	10 (33.3)	11 (47.8)	
Tested >12 months ago	9 (17.0)	6 (20.0)	3 (13.0)	
Tested within last 12 months	23 (43.4)	14 (46.7)	9 (39.1)	
Knows where to go to get a free HIV test	25 (47.2)	14 (46.7)	11 (47.8)	0.93
Has health insurance	9 (83.0)	5 (16.7)	4 (17.4)	1.00
Saw healthcare provider in last 12 months	27 (50.9)	14 (46.7)	13 (56.5)	0.48
Medical care within last 12 months				0.72
Received or did not need	27 (50.9)	16 (53.3)	11 (47.8)	
Needed and did not receive (due to cost)	17 (32.1)	10 (33.3)	7 (30.4)	
Needed and did not receive (due to other reasons)	9 (17.0)	4 (13.3)	5 (21.7)	
Mental health				
Currently depressed	33 (62.3)	20 (66.7)	13 (56.5)	0.45
Suicidality				0.70
Never thought about or attempted	20 (37.7)	12 (40.0)	8 (34.8)	
Suicidal ideation	11 (20.8)	5 (16.7)	6 (26.1)	
Attempted suicide	22 (41.5)	13 (43.3)	9 (39.1)	
Gender affirmation				
Ever used hormones for gender confirmation	25 (52.8)	17 (56.7)	8 (34.8)	0.11
Ever had any gender confirmation surgeries	10 (18.9)	6 (20.0)	4 (17.4)	1.00
Accepts body despite flaws	17 (32.1)	8 (26.7)	9 (39.1)	0.34
Comfort with gender				0.25
Very/somewhat comfortable	26 (49.1)	16 (53.3)	10 (43.5)	
Uncertain	5 (9.4)	1 (3.3)	4 (17.4)	
Somewhat/very uncomfortable	22 (41.5)	13 (43.3)	9 (39.1)	
Open (out) as a transgender individual in social/personal life				0.34
Not at all	4 (7.6)	2 (6.7)	2 (8.7)	
A little bit/some/mostly	34 (64.2)	17 (56.7)	17 (73.9)	
Completely	15 (28.3)	11 (36.7)	4 (17.4)	
Open (out) as a transgender individual at work or school				**0.02**
Not at all	14 (26.4)	5 (16.7)	9 (39.1)	
A little bit/some/mostly	19 (35.9)	9 (30.0)	10 (43.5)	
Completely	20 (37.7)	16 (53.3)	4 (17.4)	
Stigma and discrimination due to transgender identity/presentation				
Experienced any discrimination	52 (98.1)	30 (100.0)	22 (95.7)	0.43
Family not at all supportive	36 (67.9)	23 (76.7)	13 (56.5)	0.12
Has been arrested	17 (32.1)	13 (43.3)	4 (17.4)	0.07
Had problems getting health/medical services	17 (32.1)	11 (36.7)	6 (26.1)	0.41
Trauma	23 (43.4)	12 (40.0)	11 (47.8)	0.57
Has been physically abused	36 (67.9)	21 (70.0)	15 (65.2)	0.71
Has been sexually abused	26 (49.1)	11 (36.7)	15 (65.2)	**0.04**

Bold indicates *p*-value≤0.05.

Almost all participants (92%) reported having had receptive anal intercourse with a man in the last three months, most of whom (61%) had had CRAI (56% of total study sample).

### Sexual behaviour

The median age of sexual debut with a male was 13 years; this average did not differ between those who reported CRAI and those who did not. Thirty-six percent of participants were in a committed relationship at the time of data collection, all of whose committed relationships were with men; relationship status was not found to be significantly associated with CRAI. Similarly, recent insertive anal intercourse with a man (reported among 36% of the sample) and CRAI were not found to be correlated. Only one participant reported having had sex with a woman in the past three months; therefore, this variable was not included in the analysis. The majority of participants (66%) reported engaging in sex for income. Sex work was found to be strongly associated with having had CRAI in the past three months (*p=*0.01).

### HIV testing and healthcare access

Forty-three percent of participants had been tested for HIV within the last 12 months, whereas 40% had never been tested. Almost half (47%) of the sample reported knowing where to get a free HIV test. While half (51%) of the participants had been seen by a healthcare professional in the last 12 months, almost a third (32%) reported having a need for medical care in the last 12 months but being unable to obtain it, because they were unable to afford it. Only 17% of participants had health insurance. None of these variables related to HIV testing or healthcare access were found to be associated with CRAI.

### Gender affirmation

Nineteen percent of the sample reported having had gender confirmation surgeries, whereas half had ever used hormones for medical transition. One third (32%) of the sample population reported being accepting of their bodies. Regarding comfort with gender, half of the participants reported being “very” or “somewhat comfortable” while the other half were “uncertain” (9%) or “somewhat” to “very uncomfortable” (42%). Participants reported a range of degrees of being “out” or “openness” about their transgender identities in private and professional life. While the majority (64%) of participants reported being “somewhat ‘out’” in their personal lives, the sample was more divided in terms of the workplace or school, with 38% “completely ‘out’” and 26% “not at all ‘out’ or ‘open’” about being transgender. Participants who reported CRAI were significantly more likely to report being “completely ‘out’” at the workplace or school than those who reported not having had CRAI (53% vs. 17% respectively, *p*=0.02).

### Stigma and discrimination

An overwhelming majority (98%) of participants reported having experienced a form of discrimination due to their transgender identity or gender presentation in the last year; in fact, only one individual in the sample reported not having experienced discrimination. Two thirds (67%) of the sample reported family not being at all supportive of their transgender identity. One third of participants had been arrested because of being transgender or gender presentation. One third also reported being discriminated against in a medical setting for these reasons. Of these discrimination variables, a history of arrest was the only one significantly correlated with CRAI (*p*=0.07).

### Trauma

Having experienced trauma was common within the sample. Two thirds of participants reported having been physically abused or beaten because of their gender identity or presentation. A history of physical abuse, however, was not correlated with CRAI. Half (49%) of participants reported having been sexually abused or assaulted in their lifetime. A history of sexual abuse/assault, surprisingly, was found to be *negatively* associated with CRAI; those engaging in CRAI reported sexual abuse/assault less than those who did not (*p*=0.04).

### Mental health

Mental health results are reported elsewhere [23]. In the present analysis, depression, suicide ideation and suicide attempts were not significantly associated with CRAI.

### HIV Prevalence and sexual risk behaviour

Three participants reported living with HIV and an additional one participant tested positive for HIV in the rapid antibody test, administered as an optional part of the study following survey completion. Thirteen participants declined the HIV test. Thus, four participants were HIV positive out of the 40 participants (10%) who were tested as part of the study or via self-report.

### Risk behaviour associations

After conducting χ^2^ tests on the association of each of the characteristics with CRAI, a bivariate regression analysis was used to further examine the statistically significant (*p<*0.05) correlates and those with high prevalence in the study sample, in order to estimate the size of the effect of these factors on the outcome, and the degree of precision with which they were measured. In the logistic regression analysis, statistical significance was set at *p*≤0.05 to determine associations. Having sex for income was associated with a fourfold odds of having had CRAI [OR (95% CI)=4.36 (1.30, 14.67)]. Being “open”/“out” as a transgender individual at work or school was associated with having seven times the odds of having had CRAI compared to those who were not at all out [OR(95% CI)=7.20 (1.52, 33.85)]. Having a history of arrest was associated with over three times the odds of having had CRAI [OR (95% CI)=3.63 (0.99, 13.30)]. Sexual abuse/assault was negatively correlated with CRAI [OR (95% CI)=0.31 (0.10, 0.96)].

The four variables found to be associated with CRAI in the bivariate analysis were entered simultaneously into a multivariable regression model. When controlling for the other variables, having been arrested was no longer significantly associated with CRAI. A Fisher's exact test of association found no association between history of arrest and engaging in transactional sex (*p*=0.123). History of sexual abuse/assault remained negatively correlated with CRAI [OR (95% CI)=0.22 (0.53, 0.89)]. Sex for income was no longer statistically significantly associated (*p*=0.06) with CRAI [OR(95% CI)=4.05 (0.97, 16.95)]. Being completely “open”/“out” as transgender at work or school was associated with almost six times the odds of having had CRAI. Table 2 summarizes the bivariate and multivariable analyses.

**Table 2:** Factors independently associated with CRAI

	Bivariate		Multivariable	
		
	*N*=53		*N*=53	
		
Characteristic	Odds ratio (95% CI)	*P*	Odds ratio (95% CI)	*P*
Highest education level				
Did not complete high school	Ref			
Completed high school	0.28 (0.07, 1.05)	0.06	–	–
Completed some/graduated university	0.63 (0.14, 2.85)	0.55	–	–
Sex for income	4.36 (1.30, 14.67)	**0.01**	4.05 (0.97, 16.95)	0.06
Monthly income >$1000 USD	1.97 (0.57, 6.82)	0.28	–	–
Open (out) as a transgender individual at work or school				
Not at all	Ref		Ref	
A little bit/some/mostly	1.62 (0.39, 6.68)	0.50	1.43 (0.28, 7.32)	0.66
Completely	7.20 (1.53, 33.85)	**0.01**	5.97 (1.09, 32.81)	**0.04**
Family not at all supportive of transgender identity	2.53 (0.78, 8.24)	0.12	–	–
Has been arrested because of transgender identity/presentation	3.63 (0.99, 13.30)	**0.05**	2.95 (0.61, 14.41)	0.18
Has been sexually abused	0.31 (0.10, 0.96)	**0.04**	0.22 (0.53, 0.89)	**0.03**

Bold indicates *p*-value≤0.05.

## Discussion

In this sample of mostly young, Lebanon-born trans feminine individuals in Beirut, HIV prevalence (10%) was higher than that of MSM (1.5–3.6%) [24,25], but lower compared to prevalence rates among transgender women in other settings around the globe [3]. This prevalence rate among trans feminine individuals in Lebanon, as compared to other contexts, may be lower due to sample size or a lack of representativeness of our sample. The prevalence may have also been higher if more or all participants had received an HIV test as part of the study, as 13 participants declined to be tested (either because of a previous positive test or for other reasons not specified by participants). High rates of CRAI with male partner(s) in the last three months (57%) were reported, suggesting high risk for HIV transmission. Of these participants reporting CRAI, 40% reported not knowing the HIV status of the partner(s), further indicating the potential for risk.

One third (66%) of the participants reported engaging in sex work. This percentage is very similar to rates found in the United Sates [26]; engagement in sex work is often the result of economic hardship due to transphobia and discrimination [27,28]. Despite the risks associated with sex work, 40% of our sample had no history of HIV testing, which is comparable to counterparts in other countries [12,13].

Trauma such as sexual abuse/assault were common among the participants in our sample; almost half (49%) of the participants reported having experienced such trauma. Rates of having experienced physical violence (68%) and police arrest (32%) because of gender identity or presentation were also high, and all but one (98%) reported discrimination due to gender identity or presentation. We were surprised to find that in the regression analysis that a history of sexual abuse/assault remained *negatively* correlated with CRAI [OR (95%CI)=0.22 (0.53, 0.89)]. While these results could have been the result of sample size and seem to indicate that having been sexually abused reduces the likelihood of CRAI, these findings are unsupported theoretically or by data from other countries. There is no obvious reason why this association would differ in Lebanon as other studies demonstrate a positive correlation or no significant association between a history of sexual abuse/assault and sexual risk behaviour or HIV status [27,29]. Thus, these results require further exploration to determine whether sampling strategies played a role in this result or if perhaps this population has learned tools of resilience and empowerment or developed other related strengths that impact condom use negotiation and/or decision-making as the result of past experiences with sexual abuse/assault.

Not surprisingly given data from other settings, sex for income was correlated with CRAI; having transactional sex was associated with almost six times the odds of having had CRAI. This association found in Beirut is higher than that of a population of transgender women in San Francisco [30], but this correlation was not significant in the multivariate analysis, perhaps due to sample size. In some settings, in addition to the power imbalance created by transactional sex, deferring to male partners for condom use decisions has been found to be common among transgender women due to traditional patriarchal gender roles [31]. Whether these kinds of cultural influences play a stronger role in the context of sex work in Lebanon should be explored in future research. Understanding the determinants of sex work will be critical for informing future sexual health intervention strategies with this population.

Another main finding, that requires further exploration and interpretation, was that being completely “out” or “open” about transgender status at work or school was independently and significantly associated with having had CRAI, even after adjustment. Potentially related to being completely “out” or “open” at work or school, some of the variables related to Gender Affirmation may help shed some light on these results. Overall, discomfort with gender identity was common among the participants, and most were not “out” as transgender in their personal or professional lives. Although half of the participants had used hormones for medical transition, less than a quarter had had any type of gender confirmation surgeries. Most expressed dissatisfaction with their physical appearance. Further inquiry is needed to determine the impact of factors potentially related to medical transition and “outness” such as access to medical transition services, affordability of transition-related healthcare, and quality and availability of trans-friendly providers. Indeed, the aforementioned qualitative study that was conducted among trans feminine individuals in Lebanon [18] found that some participants felt that in order to be able to maintain relationships with family members, dressing “as a woman” and having breast augmentation surgeries were not an option. Further research is required to understand the potentially complex relationship between medical transition, body satisfaction, “outness” and CRAI.

## Future directions

These results contribute to the existing body of literature on HIV risk and prevalence among trans feminine individuals around the globe. Further, they highlight the need for culturally relevant strategies to mitigate the impact of HIV, stigma and transphobia in diverse international settings, and for the first time in MENA. First, the importance of trans feminine individuals’ inclusion in HIV research and programming cannot be overemphasized. Second, efforts to include trans feminine individuals in research, programming, outreach and services must distinguish between MSM and trans feminine individuals. Research efforts and funding opportunities, both in MENA and in other locations throughout the world, must recognize and prioritize the health needs of this population. Future research will explore the feasibility and acceptability of culturally adapting HIV prevention interventions to the Lebanese and Middle Eastern contexts.

## Conclusions

To our knowledge, this is the first quantitative study to measure sexual risk and HIV prevalence among trans feminine individuals in MENA. These results illuminate important health challenges that members of this understudied population face and provide the first measurements of HIV prevalence and risk behaviour. As in studies from other regions, the results of this study indicate that there is a high prevalence of HIV risk behaviour, particularly sex work and CRAI, among the trans feminine population in Lebanon. The data from this study suggest that understanding the motivations for engaging in sex work and CRAI as well as determining the potential factors that facilitate condom use are important next steps for addressing the health disparities and mitigating the effects of an emerging epidemic among trans feminine people in Lebanon.
